# The Role of Intravascular Ultrasound in the Evaluation and Treatment of Free-Floating Stent Struts Following Inadequate Ostial Circumflex Stenting: A Case Report

**DOI:** 10.3390/medicina60101563

**Published:** 2024-09-24

**Authors:** Milorad Tesic, Djordje Mladenovic, Vladan Vukcevic, Dario Jelic, Dejan Milasinovic

**Affiliations:** 1 Clinic for Cardiology, University Clinical Center of Serbia, 11000 Belgrade, Serbia; mladendjolem@gmail.com (D.M.); vladan.vukcevic@gmail.com (V.V.); duga13@gmail.com (D.J.); milasin.d18@gmail.com (D.M.); 2 Faculty of Medicine, University of Belgrade, 11000 Belgrade, Serbia

**Keywords:** left main PCI, IVUS, free-floating struts, bifurcation

## Abstract

Introduction: Excessive stent strut protrusion in the distal left main (LM) from either the left anterior descending (LAD) or circumflex (Cx) artery following inadequate ostial stenting may complicate any later procedure involving the left coronary artery. In such case scenarios, intravascular ultrasound (IVUS) guidance provides accurate assessment of the ostial stent position and may facilitate subsequent management strategies and treatment. Case summary: We present a complex percutaneous coronary intervention (PCI) of LM bifurcation in a 49-year-old man following inadequate ostial Cx stenting that resulted in excessive stent protrusion in the distal LM segment, accompanied by a subsequent short 80–90% ostial LAD stenosis. Initially, IVUS was performed to confirm “floating struts” from a previous Cx ostial stenting and to ensure complete intraluminal placement of the wire within the stent leading to the Cx, precluding any side passage through the stent struts. Then, a second wire was inserted into the LAD through the most distal stent strut under live IVUS guidance. Further PCI was completed according to the principles of the double kissing mini-culotte technique. Final IVUS runs confirmed correct stent apposition and expansion in the LM, LAD and Cx segments. Conclusions: In cases involving the treatment of “free-floating” struts in the distal LM artery, intravascular imaging is essential to ensure optimal PCI outcomes.

## 1. Introduction

Treating lesions in the ostial segments of the left anterior descending artery (LAD) or circumflex artery (Cx) poses a distinctive challenge for interventional cardiologists. Stenting solely within ostial lesions can result in either an “ostial miss” or “main branch protrusion”, increasing the risk of stent thrombosis and restenosis [1,2]. Excessive stent protrusion into the left main (LM) also further complicates any later procedure involving the left coronary artery due to increased difficulty of wiring the LAD and Cx through the correct stent struts. In such case scenarios, intravascular imaging-guided percutaneous coronary intervention (PCI) provides accurate assessment of the ostial stent and wire positions and may facilitate subsequent management strategies and treatment [3]. In everyday clinical practice, selection of a preferred imaging modality should reflect the operator’s experience and the primary objective of the assessment. Thus, in this case, the benefits of intravascular ultrasound (IVUS) over optical coherence tomography (OCT) include real-time imaging, which ensures the operator can observe the wire’s position throughout the rewiring process of both the protruded stent and later the side branch. Additionally, IVUS provides better visualization of large vessels like the LM and requires lower doses of contrast media [1,3]. However, there have been no reports on the usefulness of IVUS imaging for the assessment and treatment of excessive stent protrusion into the distal LM. Therefore, this case underscores the fundamental role of IVUS imaging guidance for LM PCI following inadequate ostial Cx stenting.

## 2. Case Report

**Figure 1:**
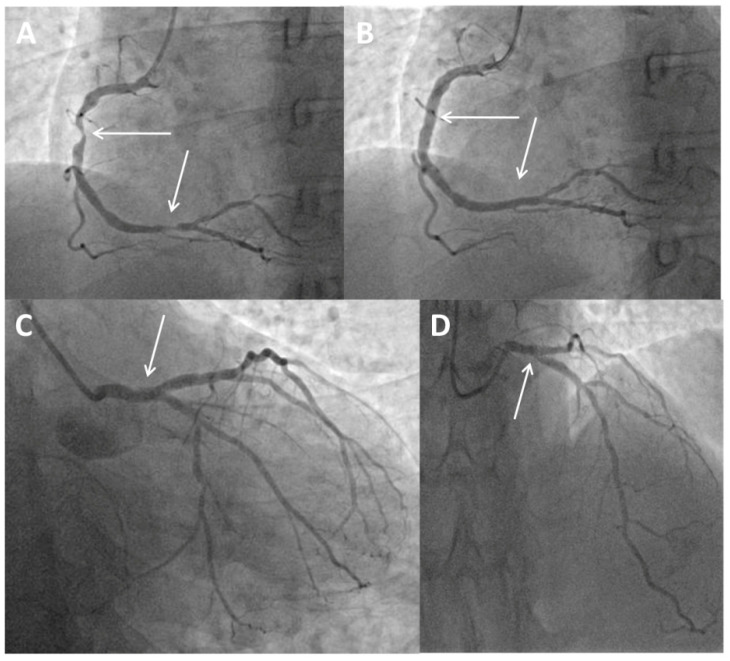
Coronary angiogram and primary percutaneous coronary intervention. (**A**) Baseline angiography showing subocclusive stenosis of the proximal and distal segments of the right coronary artery (arrows). (**B**) Final angiography showing the stented proximal and distal segments of the right coronary artery (arrows). (**C**) Protrusion of a previously implanted stent from the circumflex coronary artery to the left main (arrow). (**D**) Short, significant ostial stenosis of the left anterior descending coronary artery (arrow).

**Figure 2:**
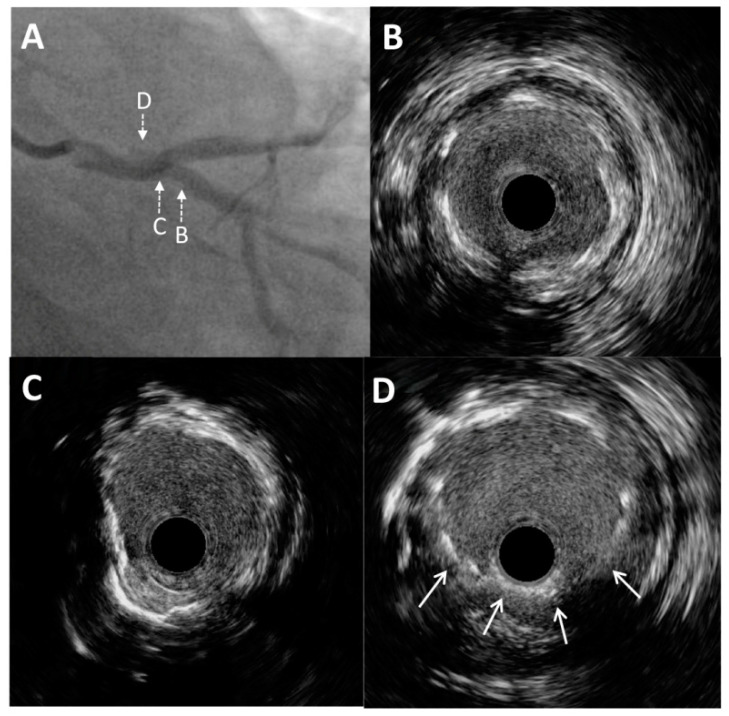
Intravascular ultrasound imaging of the left main and circumflex coronary artery. (**A**) Enlarged angiographic view of excessive stent protrusion from the circumflex artery to the left main. Pre-intervention cross-sectional intravascular ultrasound images from the proximal (**B**) and ostial (**C**) parts of the circumflex artery without significant restenosis and mild neointimal hyperplasia of a previously implanted stent. (**D**) Visualization of “floating struts”—excessive stent protrusion from the circumflex coronary artery into the left main (arrows).

A 49-year-old man presenting with chest pain and elevated high-sensitivity troponin levels was referred for coronary angiography due to suspected non-ST-segment elevation myocardial infarction. He underwent previous primary PCI of the ostial segment of the Cx using a Xience Alpine stent 3 × 15 mm (Abbott Vascular, Santa Clara, CA, USA) following a posterior ST-segment myocardial infarction three years ago. Now, coronary angiography revealed subocclusive stenosis of the proximal and distal segments of the right coronary artery (Figure 1A), which were stented with two drug eluting stents (Figure 1B). On the LM coronary artery, a suspected protrusion of a previously implanted stent from the Cx was observed (Figure 1C and Figure 2A), accompanied by a subsequent short 80–90% ostial LAD stenosis (Figure 1D). During the same hospitalization, the patient was presented to the Heart team. Since the patient had excessive stent strut protrusion in the LM that needed correction as well as significant ostial LAD stenosis, it was decided to perform an imaging-guided PCI of the LM coronary artery.

**Figure 3:**
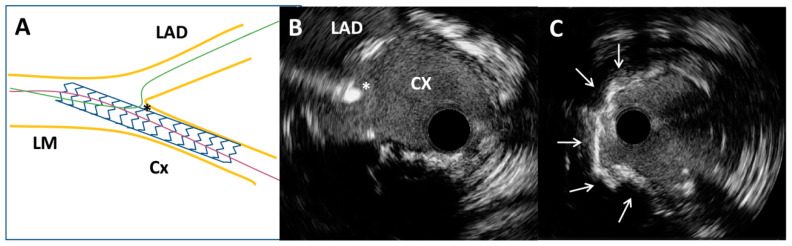
Intravascular ultrasound-guided wiring of the left anterior descending artery. (**A**) Schematic representation of the circumflex coronary artery stent and the left anterior descending coronary artery wire position (asterisk). (**B**) Intravascular ultrasound-guided wiring of the left anterior descending artery through the most distal stent strut of a previously implanted stent (asterisk). (**C**) Superficial calcium (arrows) of the ostial segment of the left anterior descending artery after high-pressure predilatation with a non-compliant balloon.

**Figure 4:**
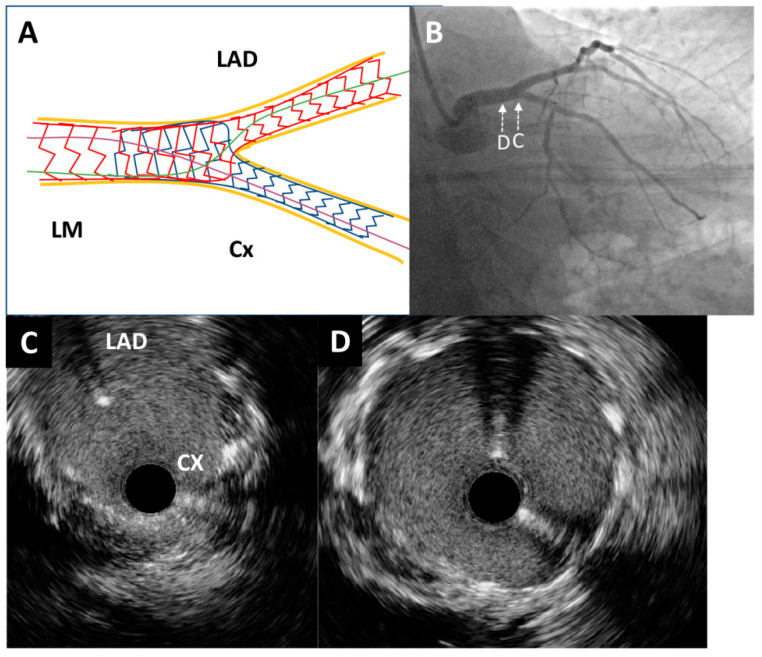
Post-procedure angiography and intravascular ultrasound imaging. (**A**) Schematic representation of the applied double kissing mini-culotte technique. (**B**) Excellent angiographic result of left main bifurcation (caudal projection). An intravascular ultrasound pullback was conducted from the circumflex artery, which showed optimal stent expansion without malapposition at both the left main bifurcation site (**C**) and the distal portion of the left main artery (**D**).

**Figure 5:**
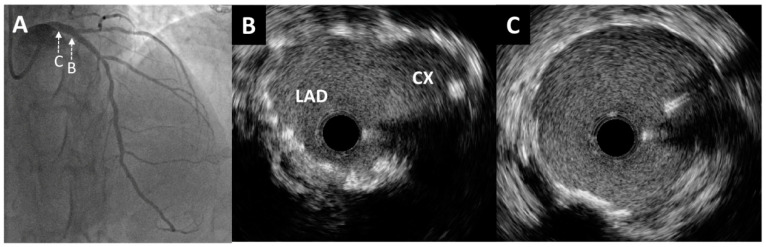
Post-procedure angiography and intravascular ultrasound imaging. (**A**) Excellent angiographic result of left main bifurcation (cranial projection). An intravascular ultrasound pullback from the left anterior descending artery showed optimal stent expansion without malapposition at both the left main bifurcation site (**B**) and the distal portion of the left main artery (**C**).

Through the radial artery, the LM was cannulated with a 6F guiding catheter. First, the Cx artery was wired and IVUS imaging with an OptiCross catheter (Boston Scientific, Natick, MA, USA) was performed. It was of utmost importance to prove with IVUS the complete intraluminal placement of the wire within the stent in the Cx artery, precluding any side passage through the stent struts. Moreover, IVUS imaging revealed that proximal (Figure 2B) and ostial (Figure 2C) parts of the Cx were without significant restenosis with mild neointimal hyperplasia of a previously implanted stent. However, there was an excessive protrusion of “floating struts” from the Cx into the distal LM (Figure 2D). Following imaging, PCI was performed according to the principles of the stepwise double kissing (DK) mini-culotte technique [4,5]. A dual lumen catheter was inserted into the Cx, followed by the placement of a second wire within the Cx. Wiring of the LAD through the most distal stent strut was conducted under live IVUS guidance (Figure 3A,B). After initial strut opening toward the LAD using a 2.0 × 15 mm semi-compliant balloon, predilation of the ostial LAD using a 3.5 × 15 mm non-compliant balloon inflated up to 19 atmospheres was attempted. However, full balloon expansion could not be achieved. Then, IVUS imaging revealed moderate to severe calcification (around 180-degree calcium arc) of the ostial LAD (Figure 3C) and distal LM at the site of the undilatable stenosis. The distal and proximal reference segments were selected, and the mean external elastic membrane diameters of the proximal LAD and LM were measured (4.35 mm and 5.46 mm, respectively). The length of the lesion was around 19 mm from the ostium of the LM to the proximal part of the LAD. Consequently, intravascular lithotripsy (IVL) was performed using a 4.0 × 12 mm lithotripsy balloon (Shockwave Medical, Inc. Santa Clara, CA, USA) inflated to four atmospheres at the distal LM and proximal LAD artery, administering a total of 50 pulses. This process was repeated until complete balloon inflation was achieved for optimal vessel preparation. After IVL, balloon “kissing” was performed using two 3.5 × 15 mm non-compliant balloons positioned toward the Cx and LAD. According to the principles of the stepwise DK mini-culotte technique (Figure 4A), an Everolimus eluting Synergy 4.0 × 24 mm stent (Boston Scientific Corporation, Marlborough, MA, USA) was implanted from the ostium of the LM into the proximal LAD, after which proximal optimization was performed using a 5.0 × 8 mm non-compliant balloon. Wiring of the Cx was completed following kissing balloon inflation using a 3.0 × 15 mm non-compliant balloon positioned toward the Cx and a 4.0 × 12 mm non-compliant balloon positioned toward the LAD. Final high-pressure (27 atmospheres) proximal optimization of the stents in the LM was performed with a 5.0 × 8 mm non-compliant balloon. Final angiography showed very good results (Figure 4B and Figure 5A), confirmed by final IVUS pullbacks from both the Cx (Figure 4C,D) and LAD (Figure 5B,C) that showed sufficient minimum stent area of the LM, LAD and Cx and complete stent expansion and apposition in both vessels without residual edge dissection. There were no periprocedural complications or adverse events during hospitalization, and the patient was discharged the next day and was without any symptoms at the one-year follow-up visit.

## 3. Discussion

This case report utilized IVUS imaging at various stages throughout the PCI procedure to determine appropriate treatment in order to reduce and prevent the occurrence of major adverse cardiac events after inappropriate stent implantation. Initially, IVUS was performed to confirm “floating struts” from a previous Cx ostial stenting and to ensure complete intraluminal placement of the wire within the stent leading to the Cx, precluding any side passage through the stent struts. Also, we demonstrated the use of live IVUS guidance to assist in rewiring the side branch (LAD artery) during LM bifurcation PCI. Furthermore, IVUS imaging provided LM bifurcation anatomy in detail to accurately measure vessel diameters, stenosis severity and lesion length and to assess lesion characteristics and the extent of circumferential calcium disease in the distal LM and ostial LAD, which prompted the use of IVL to prepare the lesion for stenting. Final IVUS runs confirmed correct stent placement and expansion in the LM, LAD and Cx segments and excluded stent malapposition which is associated with midterm stent failure [6].

The optimal technique for treating ostial lesions, particularly in the case of the ostial Cx artery, remains a topic of debate. Historically, positioning the stent at the edge of the ostium has been a commonly utilized approach for treating isolated ostial lesions of both the LAD and Cx arteries [7,8]. However, this technique carries inherent risks, including the potential for geographical miss, plaque shift or excessive protrusion of the stent struts into the LM artery. Additionally, intracoronary imaging studies have revealed that in cases where the angle between the LM and the Cx is more acute <70° (as in this case), stent adjustment to the ostium without floating struts within the LM becomes impossible. Therefore, crossover stenting (covering the affected ostial LAD or ostial Cx along with the diseased segment of the LM) followed by proximal optimization technique and eventual kissing is regarded as a preferable option [1].

In this particular case, since the patient had excessive stent strut protrusion in the LM that needed correction as well as significant ostial LAD stenosis (low residual SYNTAX Score I-12), it was decided to perform an imaging-guided PCI of the LM coronary artery according to the principles of the stepwise DK mini-culotte technique [4,5]. In bench tests, the DK mini-culotte technique resulted in significantly lower stent malapposition in at least one location of the bifurcation compared to both the standard culotte and DK crush techniques [4]. Furthermore, in the DK mini-culotte stenting models, it was shown that with additional kissing balloon dilation, side branch stents and their cells were more fully expanded, the side branch stents had better apposition and the issues of underexpansion, malapposition, and mal-distribution observed in standard culotte stenting were significantly improved [4]. Consequently, a conducted clinical trial reported lower incidence rates of major adverse cardiovascular events (MACE) (4.55% vs. 13.6%) and restenosis (1.52% vs. 12.12%, *p* = 0.033) in the DK mini-culotte technique in comparison to provisional T stenting at the one-year follow-up [9]. Furthermore, a recent study showed a significantly lower cumulative event rate of MACE at 5 years (8.3% vs. 22.8%, *p* = 0.007) and restenosis (6.3% vs. 17.7%, *p* = 0.018) in the DK mini-culotte technique in comparison to the standard mini-culotte technique [10]. Results of these studies, which were based on the treatment of complex bifurcation lesions, suggest that the technical improvements in the DK mini-culotte technique are safe and effective. However, the clinical relevance of these improvements needs to be confirmed in a larger in vivo study population, particularly in comparison to the DK crush technique, which is currently widely performed. The main limitation of the culotte technique is the part where both stents that were used to cover branches also have to cover and overlap in the main bifurcation vessel [9]. Consequently, culotte may not be the optimal technique for bifurcation lesions with very different branch sizes [9]. Also, attention is required to select stent platforms that can be safely expanded to reach the size of the proximal main bifurcation vessel. In comparison to other stent techniques, both the DK mini-culotte and DK crush techniques can be technically challenging and time-consuming. This can increase the overall cost of the procedure due to extended use of resources in the catheterization laboratory, including physician and staff time as well as equipment use.

The introduction of the newly released high-definition IVUS represents a significant advancement in IVUS technology. This innovative system merges high-resolution imaging with enhanced image depth, making it an appealing evolution in the field. Thus, IVUS-guided PCI may avoid the limitations of angiographic ambiguity in visualization of lesions and help to understand the causes of stent failure after previous PCI. Furthermore, trials suggest that with IVUS or OCT guidance, PCI is superior to angiographic guidance in terms of death, myocardial infarction, target lesion revascularization, in-stent revascularization and stent thrombosis, even in populations with complex coronary lesions [11,12,13,14]. Even though OCT has nearly 10 times higher axial resolution in comparison to IVUS [14], the benefit of IVUS over OCT is that it can be used in real time, which allows the operator to be certain of the wire’s position at all times during the rewiring process, as in this case. Also, IVUS is preferable in visualization of ostial lesions and large vessels such as the LM. Another benefit of IVUS over OCT is that there is no requirement for contrast media injection to obtain a correct image. This allows live IVUS guidance to be used in patients who require lower doses of contrast media injection due to other conditions.

With respect to the known imaging capabilities of IVUS and OCT regarding resolution and penetration depth, operators should select the imaging tool based on the clinical need, anatomic segment of interest, angiographic appearance of the vessel, lesion characteristics and operator experience. Since IVUS has a lower resolution in comparison to OCT, it may be a limiting factor when more detailed evaluation is needed, e.g., superficial plaque assessment or suboptimal PCI result identification (underexpansion, strut malapposition, residual coronary edge dissections or plaque prolapse) [15]. However, the accuracy and quality of IVUS imaging largely depend on the operator’s skill and experience. To mitigate this limitation, artificial intelligence and machine learning algorithms could enhance procedural success by offering automated lesion recognition and guidance for optimal stent sizing and positioning [16,17].

## 4. Conclusions

This case report underscores the decisive role of IVUS imaging in both the evaluation and treatment of “free-floating” struts following inadequate ostial circumflex stenting.

It is evident that coronary angiography alone in such cases is not sufficient to guide lesion preparation and stent deployment. Thus, in certain complex cases, intravascular imaging must be utilized to ensure optimal PCI outcomes.

## Data Availability

The data presented in this study are available on request from the corresponding author.
